# Interleukin-6, MCP-1, IP-10, and MIG are sequentially expressed in cerebrospinal fluid after subarachnoid hemorrhage

**DOI:** 10.1186/s12974-016-0675-7

**Published:** 2016-08-30

**Authors:** Aichi Niwa, Koji Osuka, Takahiro Nakura, Naoki Matsuo, Takeya Watabe, Masakazu Takayasu

**Affiliations:** Department of Neurological Surgery, Aichi Medical University, 1-1 Yazakokarimata, Nagakute, Aichi 480-1195 Japan

**Keywords:** Interleukin-6, IP-10, MCP-1, MIG, Subarachnoid hemorrhage

## Abstract

**Background:**

Interleukin-6 (IL-6), an inflammatory cytokine, plays important roles in cerebrospinal fluid (CSF) after subarachnoid hemorrhage (SAH). Chemokines are chemoattractant cytokines that regulate trafficking of monocytes/macrophages and lymphocytes to sites of inflammation. However, no studies have been reported regarding the temporal expression of these cytokines in CSF after SAH.

**Findings:**

The concentrations of IL-6, monocyte chemoattractant protein-1 (MCP-1), interferon-γ-inducible protein-10 (IP-10), and monokine induced by interferon-γ (MIG) in the CSF of ten patients with SAH were measured using ELISA kits over a period of 14 days. All aneurysms were located in the anterior circulation. CSF samples from patients with unruptured aneurysms were used as controls. The concentration of IL-6 significantly increased during the acute stage of the disease. The concentration of MCP-1 increased from days 1 to 5, peaking on day 3, and decreased thereafter. The concentrations of IP-10 and MIG progressively increased, peaked on day 5, and then gradually decreased. There were strong correlations between the maximum levels of IL-6 and MCP-1 and IP-10 and MIG on day 5. The maximum level of IL-6 was much higher in poor outcome patients than in good outcome patients.

**Conclusions:**

The present investigation demonstrated that increases in IL-6 levels may induce the expression of MCP-1 in CSF after SAH, followed by increases in the expression of IP-10 and MIG. Dynamic changes in the levels of these cytokines may induce inflammation and may be closely associated with the development of delayed ischemic neurological deficits after SAH.

**Electronic supplementary material:**

The online version of this article (doi:10.1186/s12974-016-0675-7) contains supplementary material, which is available to authorized users.

## Findings

### Introduction

Intracisternal injections of latex beads or talc (crystallized hydrous magnesium sulfate) can induce persistent and severe cerebral vasospasm without hemorrhage, which suggests that inflammation may play an important role in inducing cerebral vasospasm [1–3]. Dramatically increased levels of IL-6, a well-known inflammatory cytokine, have been reported in CSF after SAH, a finding suggestive of a severe inflammatory response affecting the central nervous system (CNS) [4]. In the case of meningitis, the CSF concentration of IL-6 is increased [5]. Under this circumstance, patients suffering from cerebral vasospasm also have much higher CSF concentrations of IL-6 [5].

Chemokines recruit leukocytes to sites of inflammation and participate in inflammatory processes. Chemokine expression has been characterized in diseases of the CNS. Increased concentrations of monocyte chemoattractant protein-1 (MCP-1) have been reported in the CSF of amyotrophic lateral sclerosis patients, which suggests that MCP-1 plays a role in CNS inflammation [6]. In multiple sclerosis (MS), the expression of interferon-γ-inducible protein-10 (IP-10) in CSF is increased, while that of MCP-1 is decreased [7]. These chemokines are correlated with the clinical activity of MS. In the case of SAH, MCP-1 levels in CSF have been shown to correlate with cerebral vasospasm [8]. However, the time course of the expression of MCP-1 and the other abovementioned inflammatory cytokines and chemokines in CSF after SAH remains unknown. The present study was therefore performed to elucidate the serial changes in the concentrations of IL-6, MCP-1, IP10, and monokine induced by interferon-γ (MIG) in the CSF after SAH.

## Methods

### Patients and control subjects

This study included ten patients who underwent surgical obliteration of cerebral aneurysms at Aichi Medical University Hospital within 1 day of the onset of SAH. These patients, three men and seven women, ranged from 41 to 75 years of age (mean age of 57 years). Their baseline clinical characteristics are shown in Table 1. After their aneurysms were clipped, cisternal catheters were placed into their chiasmatic or prepontine cisterns, and postoperative CSF samples were collected on each of the following 14 days. One patient developed delayed ischemic neurological deficits. All the other patients had normal postoperative courses. Glasgow outcome scale was evaluated 3 months after the onset of SAH. No patients suffered from infection. CSF samples were obtained from another five patients (mean age of 62 years) who had undergone clipping for unruptured cerebral aneurysms and were used as controls. All samples were immediately centrifuged upon collection, and the supernatants were stored at −80 °C until analysis. The Ethics Committee of Aichi Medical University approved this clinical experiment.

**Table 1:** Clinical data pertaining to ten patients with subarachnoid hemorrhage

Case	Age, gender	Location of aneurysm	H & K	Fisher	Symptomatic vasospasm	The latest sampling day^a^	GOS
1	45, F	ICPC	2	3	−	14	GR
2	71, F	A. com	2	3	−	14	MD
3	53, M	MCA	3	4	+	14	VD
4	65, F	MCA	2	3	−	14	GR
5	49, M	A. com	4	3	−	14	GR
6	75, F	A. com	2	4	−	14	MD
7	41, M	A. com	2	3	−	7	MD
8	46, F	ICPC	2	3	−	10	GR
9	60, F	A. com	3	4	−	10	GR
10	64, F	A. com	4	3	−	10	MD

*H & K* Hunt & Kosnik grade, *GOS* Glasgow outcome scale, *M* male, *F* female, *ICPC* internal carotid-posterior communicating artery, *A. com* anterior communicating artery, *MCA* middle cerebral artery, *GR* good recovery, *MD* moderate disability, *VS* vegetative state

^a^The day when the last CSF was collected

### CSF analysis

The concentrations of IL-6, MCP-1, IP-10, and MIG were measured using enzyme immunoassays (EIA; R&D Systems, Inc., Minneapolis, MN, USA). The detection limits of the assays were 0.70 pg/ml for IL-6, 1.7 pg/ml for MCP-1, 1.67 pg/ml for IP-10, and 3.84 pg/ml for MIG.

### Statistical analysis

Data are expressed as the mean ± SEM. Significant differences between groups were assessed using one-way ANOVA, followed by Fisher’s PLSD for multiple comparisons. Correlations between cytokine and chemokine levels were assessed by Pearson’s correlation coefficient. Significant differences between groups were assessed by the Mann-Whitney *U* test. Significance was set at *p* < 0.05.

## Results

### Changes in IL-6, MCP-1, IP-10, and MIG levels after SAH

**Fig. 1:**
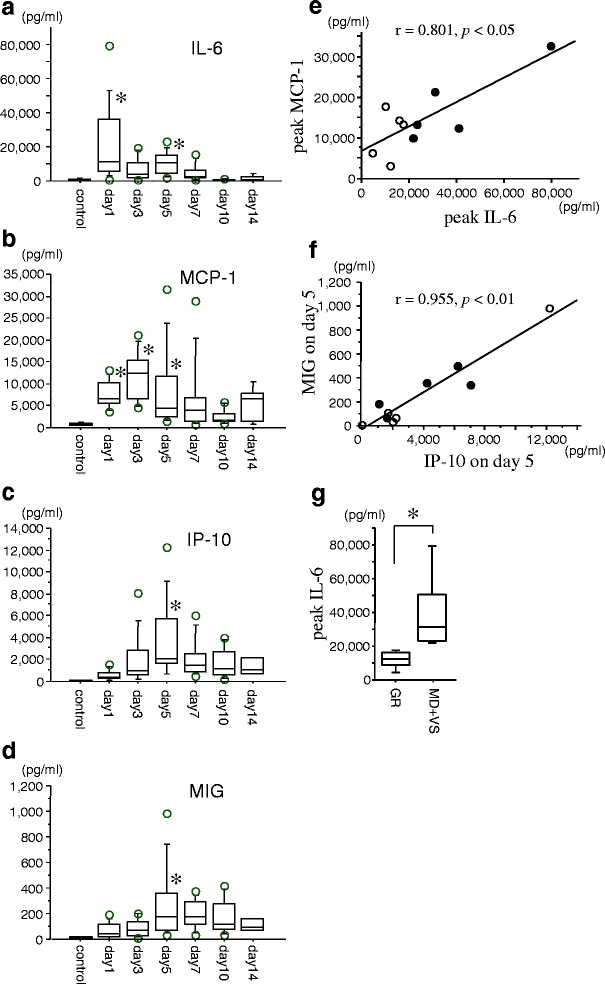
Dynamic changes in the levels of interleukin-6 (IL-6; **a**), monocyte chemoattractant protein-1 (MCP-1; **b**), interferon-γ-inducible protein-10 (IP-10; **c**), and monokine induced by interferon-γ (MIG; **d**) in the cerebrospinal fluid of ten patients with subarachnoid hemorrhage. Data are represented as box-and-whisker plots indicating the median, the upper and lower quartiles, and the largest and the lowest values. *p < 0.05 compared with control group via one-way ANOVA, followed by Fisher’s PLSD for multiple comparisons. The correlations between the peak concentration of IL-6 and that of MCP-1 (**e**) and the concentrations of IP-10 and MIG on day 5 (**f**), according to Pearson’s correlation coefficient. The *open circle* represents the data from the good outcome (good recovery (GR)) patients, while the *closed circle* represents the data from the poor outcome (moderate disability (MD) + vegetative state (VS)) patients. Peak concentrations of IL-6 (**g**) in the good outcome (GR) patients and poor outcome (MD + VS) patients. *p < 0.05 by Mann-Whitney *U* test

The concentration of IL-6 in the CSF of patients with unruptured aneurysms was 9.7 ± 1.4 pg/ml. The concentration of IL-6 increased significantly during the acute stage of the disease, reaching a level more than 1000 times that of the control group, and then decreased gradually thereafter (Fig. 1a). Following the increase in the concentration of IL-6, the concentration of MCP-1 increased from days 1 to 5, peaking at day 3, and then decreased thereafter (Fig. 1b). The concentrations of IP-10 and MIG increased progressively, peaked on day 5, and then decreased gradually thereafter (Fig. 1c, d). The concentrations of MCP-1, IP-10, and MIG in the CSF of patients with unruptured aneurysms were 732.0 ± 133.2 pg/ml, 64.2 ± 7.8 pg/ml, and 9.1 ± 3.0 pg/ml, respectively.

The peak concentration IL-6 was positively correlated with that of MCP-1, according to Pearson’s correlation coefficient (*r* = 0.801, *p* < 0.05, Fig. 1e). However, no other correlations were noted with respect to the peak concentrations of IL-6, MCP-1, IP-10, and MIG (Additional file 1: Table S1). It is intriguing that the concentration of IP-10 on day 5 was positively correlated with that of MIG on day 5, according to Pearson’s correlation coefficient (*r* = 0.955, *p* < 0.01, Fig. 1f). The peak concentration of IL-6 increased significantly in poor outcome (moderate disability (MD) + vegetative state (VS)) patients compared with good outcome (good recovery (GR)) patients (Fig. 1g), while peak concentrations of all the other chemokines showed no significance between the two groups (Additional file 2: Table S2).

## Discussion

In this study, we demonstrated that the concentration of IL-6 increased immediately after the onset of SAH, followed by an increase in MCP-1, whose level peaked on day 3, and increases in IP-10 and MIG, whose levels peaked on day 5. There were strong correlations between the maximum levels of IL-6 and MCP-1 and IP-10 and MIG on day 5.

We have previously shown that long-lasting cerebral vasospasm is induced by intracisternal injections of IL-6 in canines [9]. We have also confirmed that IL-6 induces phosphorylation of JAK1 and STAT3 in the rat basilar artery after SAH, which transduces signals into adjacent cell nuclei, resulting in transcription of acute-phase genes [10]. IL-6 is a reliable early indicator of increased vasospasm risk after SAH [11], which is in agreement with our data indicating that poor outcome patients exhibited higher IL-6 levels. IL-6 plays a role in amplifying leukocyte accumulation at sites of inflammation by activating STAT3. This is achieved in part by augmenting local production of MCP-1 and ICAM-1 [12]. MCP-1 belongs to the β or CC family of chemokines and stimulates migration of monocytes to inflammatory tissues. Previous studies have shown that activated mononuclear leukocytes can be found surrounding the major cerebral arteries in the subarachnoid space after SAH [13]. In addition, MCP-1 has been found to be significantly expressed in the major cerebral arteries in the setting of cerebral vasospasm in rats [14]. Given these findings and our data showing that a strong correlation exists between the maximum levels of IL-6 and MCP-1, immediate expression of IL-6 may play an important role in inducing MCP-1 expression via STAT3 after SAH.

IP-10 belongs to the α or CXC family of chemokines and was originally identified as an interferon-γ-inducible protein. IP-10 stimulates migration of monocytes and T cells to inflammatory tissues. However, it does not induce a chemotactic activity in neutrophils. In experimental autoimmune encephalomyelitis (EAE), astrocytes were found to secrete MCP-1 and IP-10 and to play a significant role in intrathecal inflammation [15]. MCP-1 and IP-10 are also expressed in the CSF of patients with viral meningitis and induce chemotaxis of peripheral blood mononuclear cells (PBMCs) [16]. MIG is also a member of the CXC chemokine family and activates lymphocytes. MIG and IP-10 were found to be highly expressed in the synovial tissues of patients with rheumatoid arthritis (RA) [17], which suggests that these chemokines may play an important role in the pathophysiology of RA. In the case of EAE, MIG was expressed predominantly by macrophages/microglia, while IP-10 was produced by astrocytes surrounding inflammatory lesions [18]. IP-10 and MIG have different spatial and cellular localizations in the CNS, which suggests that these chemokines have specialized functions. We found that the concentrations of IP-10 and MIG increased significantly during the first 5 days after SAH and that there was a significant correlation between the concentrations of IP-10 and MIG on day 5, which suggests that these chemokines may play a role in the development of delayed ischemic neurological deficits through simultaneous activation of monocytes and lymphocytes. More studies are needed to explore the CNS expression sites of these chemokines after SAH.

In wound healing, recruitment of various leukocyte subtypes to wound sites is essential. Using a skin wound model, macrophage migration was induced by MCP-1 expression from day 2 onward. The migration of lymphocytes was initiated by the expression of MCP-1 and was accompanied by the subsequent expression of IP-10 and MIG after 4 days [19]. This time course of chemokine expression is in agreement with our data, suggesting that the dynamic expression of chemokines and their subsequent recruitment of leukocyte subtypes play important roles in inflammation in the CNS after SAH.

In this study, we explored serial changes in the levels of inflammatory cytokines and chemokines after SAH. There were several limitations to this study. We enrolled only a small number of patients of widely varying ages and with widely varying outcomes. It is possible that these factors affected the cytokine concentrations in the CSF of these patients. Moreover, our results are purely observational, as we performed no animal experiments to corroborate our findings. Only one patient in this study suffered from symptomatic vasospasm; therefore, it is unclear whether the abovementioned cytokines play roles in the pathogenesis of delayed ischemic neurologic deficits after SAH. The sample size was not large enough to allow patient subgroup analyses. Our preliminary data confirmed only that strong correlations exist between the maximum levels of IL-6 and MCP-1 and IP-10 and MIG on day 5 after SAH. Additional studies involving larger numbers of patients are necessary to determine whether these factors are associated with the development of delayed brain injury after SAH.

## Conclusion

This study showed for the first time the dynamic changes that occur in the concentrations of inflammatory cytokines and chemokines in CSF after SAH. Our finding regarding the sequential expression of cytokines and chemokines in CSF after SAH was particularly interesting, as it suggests that these molecules may be involved in the development of delayed ischemic neurologic deficits. Especially, IL-6 was one of the factors of poor prognosis. More data are needed to determine if there is a difference in the expression levels of these chemokines between symptomatic and asymptomatic patients and to elucidate the mechanism underlying the sequential expression of these chemokines. Therapeutic interventions targeting these chemokines may provide a means of treating delayed ischemic neurologic deficits after SAH.

## Additional files

Additional file 1: Table S1. Correlation coefficients showing the relationships among the levels of interleukin-6 (IL-6), monocyte chemoattractant protein-1 (MCP-1), interferon-γ-inducible protein-10 (IP-10), and monokine induced by interferon-γ (MIG). The maximum value of each cytokine or chemokine was adopted as its peak value. (DOC 28 kb) Additional file 2: Table S2. Statistical analysis comparing the good recovery (GR) group (*n* = 5) and the moderate disability (MD) plus vegetative state (VS) group (*n* = 5) via the Mann-Whitney *U* test. (DOC 28 kb)

## Abbreviations

IL: Interleukin
CSF: Cerebrospinal fluid
SAH: Subarachnoid hemorrhage
