# Supplementary material for: Interleukin-6, MCP-1, IP-10, and MIG are sequentially expressed in cerebrospinal fluid after subarachnoid hemorrhage
Source: J Neuroinflammation. 2016 Aug 30;13(1):217. doi: 10.1186/s12974-016-0675-7 (PMC5006407; doi:10.1186/s12974-016-0675-7)
Supplement: Additional file 1: Table S1. — Correlation coefficients showing the relationships among the levels of interleukin-6 (IL-6), monocyte chemoattractant protein-1 (MCP-1), interferon-γ-inducible protein-10 (IP-10), and monokine induced by interferon-γ (MIG). The maximum value of each cytokine or chemokine was adopted as its peak value. (DOC 28 kb) [file 12974_2016_675_MOESM1_ESM.doc]

Additional file 1: Table S1

Correlation coefficients showing the relationships among the levels of interleukin-6 (IL-6), monocyte chemoattractant protein-1 (MCP-1), interferon-γ-inducible protein-10 (IP-10) and monokine induced by interferon-γ (MIG). The maximum value of each cytokine or chemokine was adopted as its peak value.

*correlation*

*coefficient p*

peak IL-6 *vs* peak MCP-1 0.801 0.035

peak IP-10 0.201 0.589

peak MIG 0.628 0.070

peak MCP-1 *vs* peak IP-10 0.445 0.205

peak MIG 0.345 0.379

peak IP-10 *vs* peak MIG 0.572 0.111
