# Supplementary material for: Interleukin-6, MCP-1, IP-10, and MIG are sequentially expressed in cerebrospinal fluid after subarachnoid hemorrhage
Source: J Neuroinflammation. 2016 Aug 30;13(1):217. doi: 10.1186/s12974-016-0675-7 (PMC5006407; doi:10.1186/s12974-016-0675-7)
Supplement: Additional file 2: Table S2. — Statistical analysis comparing the good recovery (GR) group (n = 5) and the moderate disability (MD) plus vegetative state (VS) group (n = 5) via the Mann-Whitney U test. (DOC 28 kb) [file 12974_2016_675_MOESM2_ESM.doc]

Additional file 2: Table S2

Statistical analysis comparing the good recovery (GR) group (n = 5) and the moderate disability (MD) plus vegetative state (VS) group (n = 5) via the Mann-Whitney U test.

GR MD + VS *p*

Concentration peak (pg/ml)

IL-6 11,988 ± 2,314 39,349 ± 10,624 0.009*

MCP-1 11,011 ± 2,700 17,869 ± 4,108 0.465

IP-10 3,123 ± 1,269 4,436 ± 993 0.347

MIG 130.3 ± 29.8 293.6 ± 70.7 0.117
